# A network-based group testing strategy for colleges

**DOI:** 10.1007/s41109-021-00431-1

**Published:** 2021-11-24

**Authors:** Alex Zhao, Kavin Kumaravel, Emanuele Massaro, Marta Gonzalez

**Affiliations:** 1 University of California, Berkeley, Berkeley, USA; 2 European Commission Joint Research Centre, Ispra, Italy

**Keywords:** Covid-19, Colleges, Testing

## Abstract

Group testing has recently become a matter of vital importance for efficiently and rapidly identifying the spread of Covid-19. In particular, we focus on college towns due to their density, observability, and significance for school reopenings. We propose a novel group testing strategy which requires only local information about the underlying transmission network. By using cellphone data from over 190,000 agents, we construct a mobility network and run extensive data-driven simulations to evaluate the efficacy of four different testing strategies. Our results demonstrate that our group testing method is more effective than three other baseline strategies for reducing disease spread with fewer tests.

## Introduction

Previous work in the field of network science on COVID-19 has demonstrated the positive effect that robust testing and contact tracing can have on preventing infections and keeping coronavirus cases within hospital capacity (Reyna-Lara et al. 2021; Aleta et al. 2020). Successful contact tracing and subsequent testing is, however, arduous, expensive, and requires extensive research into an individual’s contacts. To better tackle this problem, we introduce a novel method of group testing for infectious disease when limited information is known about the underlying interaction network. Our method outperforms three benchmarks by reducing disease spread while requiring fewer tests overall, reduces the attack rate and effective reproduction rate, and is robust to varying prevalence levels and other changes in parameters.

### Group testing

Governments around the world rely on testing for Covid-19 to identify and contain an outbreak. The nature of polymerase-chain reaction (PCR) testing, however, suffers from limited throughput and high cost. Group testing, a method developed to test a pool of multiple samples simultaneously, has been proposed as a strategy to increase the efficiency of testing in populations with a low prevalence rate (Gollier and Gossner 2020; Crozier et al. 2021). If implemented correctly, a group test is positive if at least one sample within the pool is positive. In minipool testing for SARS-CoV-2, nucleic acid is extracted from respiratory samples; a sample of each nucleic acid preparation is then combined into pools of samples. SARS-CoV-2 specific real-time RT-PCR is then used on the minipools. Individual testing is used for a minipool if it tests positive (Eis-Hübinger et al. 2020).

Multi-stage group testing can be defined as a variant of group testing in which the pool size *N* is divided into *x* groups and there exist *k* stages where $k > 1$. *N* = $x^{k-1}$ is the initial pool size, “which is divided by *x* in each subsequent stage, resulting in pool sizes $x^{k-1}$, $x^{k-2}, \ldots , x^0=1$ in stages $1, 2, \ldots , k$” (Eberhardt et al. 2020). In this paper, we study the two-stage group test, i.e. $k=2$. In the first stage, *n* samples are pooled and tested. If no virus is detected, then all samples are uninfected. If the pool tests positive, we proceed to the second stage where every individual is tested. The two-stage technique was successfully used in Wuhan, China where 6.5 million residents were tested within a few days (Wee and Wang 2020).

**Fig. 1:**
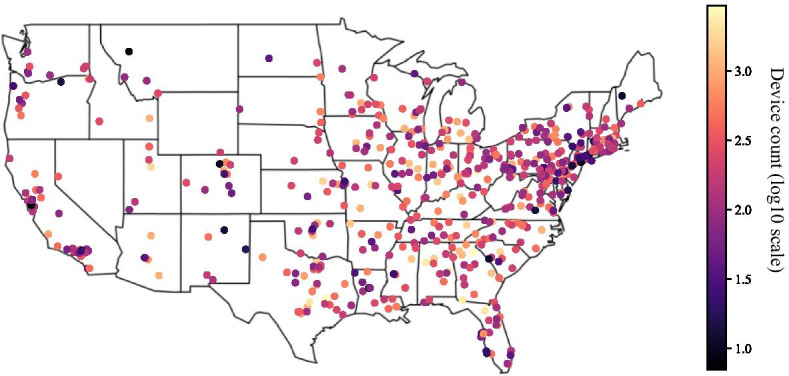
Device count and location of college clusters. Though the simulation includes a small number of agents located in Puerto Rico and Hawaii, they were excluded from this figure

### Mobility networks

Mobility networks have been used for predicting disease outbreaks (Salathé et al. 2010; Chang et al. 2021). Similar to our paper, Chang et al. (2021) construct a mobility network map individuals to places with cellphone data and run an SEIR model. The resulting simulation accurately predicts Covid-19 dynamics, such as the disproportionate effect of super-spreader events and the unequal impact of the disease on minorities. Deckert et al. (2020) apply simulations to evaluate pooled testing with homogenous groups and find substantial efficiency gains. However, we differ from Deckert et al. (2020) by focusing on college towns, initializing our simulation from mobility data, and proposing a group testing method that requires minimal contact tracing.

Group testing can also be applied in way to exploit the network structure of human interaction. Since disease spreads through interaction, outbreaks often happen within a community. Testing a group whose infections are correlated is more efficient than randomly sampling groups i.i.d. Our contribution is analyzing the efficacy of group testing strategies compared to other testing strategies in a setting where we have limited knowledge of the underlying interaction graph. In terms of resource efficiency, our method compares favorably to centrality-based strategies that rely on ranking all agents by betweenness centrality or node degree; these methods require surveying every person and recording a vast amount of data. In dense, complex environments like colleges, determining the global structure of students’ interactions is nearly impossible. Hence, we propose a testing strategy that randomly selects individuals and pools their contacts. The friendship paradox states that on average, most individuals’ friends have more friends than the individual. Cohen et al. (2003) show that vaccinating random acquaintances of randomly selected nodes is an effective immunization strategy. A social network study indicated that selecting people at random and tracing their contacts makes it possible to recognize contagion outbreaks earlier (Christakis and Fowler 2010).

**Fig. 2:**
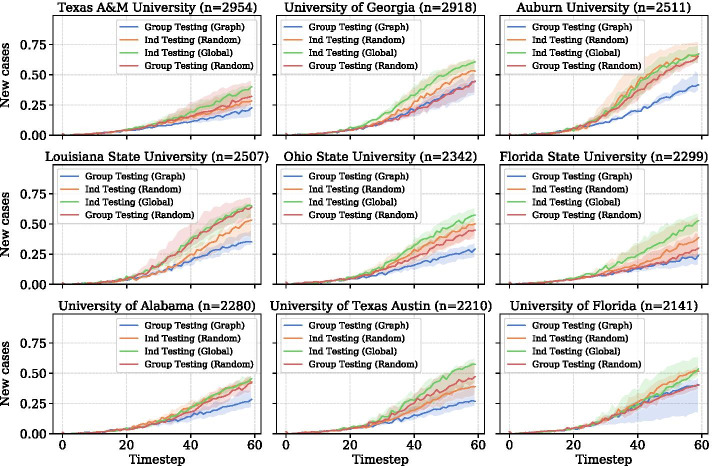
Case counts over time for the nine colleges with the largest $\texttt {device\_counts}$ (see “Appendix” for definition). The line is the median case count and the shaded areas represent the interquartile range. For all nine plots, $q=0.15$ and $\delta =0.2$

### Epidemiology at colleges

In general, college students are under the age of 30 and in the demographic with fewer risk factors for SARS-CoV-2. Many students may believe that they are less likely to be affected by the disease, and thus less compliant with shelter-in-place (Levin et al. 2020). Devising effective, cost-efficient strategies for detecting outbreaks at colleges will be crucial as schools around the country seek to reopen in the near future. College campuses are unique epidemiological environments for a few reasons. First, college students come from diverse geographical and socioeconomic backgrounds. During the semester, students normally travel frequently between campus and their hometown. Second, mobility data gathered in college towns can be assumed to be a statistically representative sample of the population, given the high degree of cell phone use. Third, college administrations can more easily observe interactions between students. Students and staff consent to a central administration logging personal records, enabling targeted contact tracing and surveillance testing.

Walke et al. (2020) find that Covid-19 cases spiked by 62.7% among those aged 18 through 22 during the arrival of students at universities for the start of the fall term. They mention that there were “more than 26,000 Covid-19 cases at more than 750 colleges across the nation by August 26... and more than 130,000 cases at 1300 colleges by September 25”. Paltiel et al. (2020) use analytic modeling of a hypothetical college student group (n=5000) to conclude that a low-sensitivity, high-specificity test occurring once every 2 days might be required for sufficient control and prevention of outbreaks. Using an SEIR model and Bayesian learning, Lu et al. (2020) find that college campuses contain a high risk of Covid-19 infections relative to nationwide averages and may have super-spreader potential for surrounding communities. They conclude that robust testing and other measures will be needed to contain spread.

## Methodology

### Data

We rely on mobile device data from SafeGraph Inc. to simulate a transmission network. SafeGraph Social Distancing Metrics record the movement of anonymized mobile devices on the census block group (CBG) level. SafeGraph’s data has already been used to construct mobility networks for studying Covid-19 (Klise et al. 2021). Previous work has also found SafeGraph data to be generally representative of the U.S. population (Chen et al. 2019). We select a single date, October 15, 2020, to initialize the network. Though any date would be mostly arbitrary, we chose October 15 because all colleges start their fall terms by October and the date is prior to fall or winter breaks.

For the sake of focusing on college towns, we select only CBGs with an undergraduate population greater than 40% as determined by the 2016 American Community Survey (ACS). Run by the U.S. Census Bureau, the ACS contacts over 3.5 million households every year to collect social, economic, and demographic information (Bureau U.C. 2017).

To analyze the distribution of devices across the country, we grouped the CBGs with an undergraduate population greater than 40% by agglomerative clustering with a distance threshold of ten miles. The resulting 533 clusters can be seen in Fig. 1. There are a concentration of smaller college towns in New England. Large colleges with more than 1000 devices in our sample tend to be in the South. Figure 2 shows the nine largest colleges by device count and their simulated case counts over time.

### Infection model

**Fig. 3:**
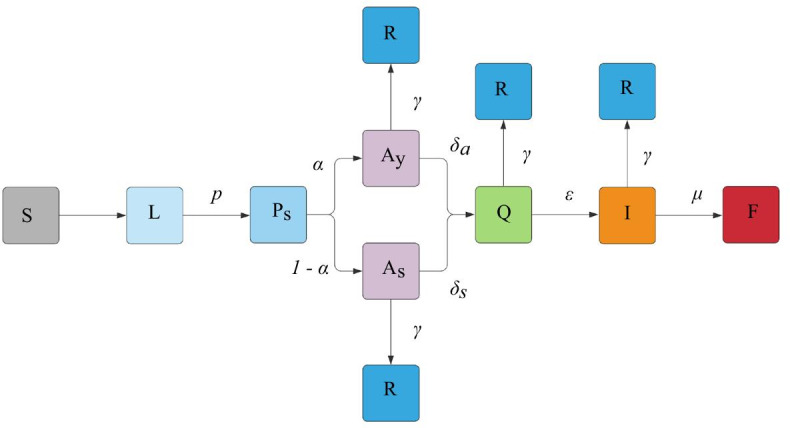
Flow diagram demonstrating states

We construct a discrete-time susceptible–latent–infected–removed (SLIR) stochastic model with the incorporation of additional states for SARS-CoV-2 transmission, as seen in Fig. 3.

Individuals begin in the susceptible (S) state. At each time step *t*, presymptomatic ($P_s$), asymptomatic($A_y$), and symptomatic($S_y$) individuals have a probability $\beta$ of infecting susceptible individuals if they come in contact. Upon initial infection, individuals enter the latent state (L) and then enter the $P_s$ state. Presymptomatic individuals have a probability $\alpha$ of entering the $A_y$ state and a ($1 -\alpha$) probability of entering the $S_y$ state. Those in the $S_y$ state then have a probability $\delta _s=\delta$ of entering the quarantine state (Q), while agents in $A_y$ have a probability $\delta _a=\frac{\delta }{2}$ of entering Q. This encapsulates the fact that asymptomatic individuals are less likely to self-quarantine, since they may not realize they have contracted the virus. $\delta _a,\delta _s$ = 1 when individuals in any state test positive. Next, individuals in Q have a probability $\varepsilon$ of going to the ICU (I) regardless of whether they entered Q from the $A_y$ or the $S_y$ state. This greatly over-simplifies the real-world disparity in outcomes for asymptomatic and symptomatic patients. However, we acknowledge this weakness; the difference between the two states in our simulation is primarily to examine the disparity in voluntary quarantining $\delta$. Individuals in I have a probability $\mu$ of dying and entering the fatal state (F). As a final addition, individuals in the $A_y$, $S_y$, Q, and I phases all have a probability $\gamma$ of recovering (R). All epidemiological parameters are contained in Table 1.

**Table 1:** Epidemiological parameters of simulation

Description	Parameter	Value	Reference
Secondary attack rate	$\beta$	0.09	Jing et al. (2020)
$L \rightarrow P_s$	*p*	1/3 $\hbox {day}^{-1}$	Backer et al. (2020)
$P_s \rightarrow A_y$	$\alpha$	0.7	Barrett et al. (2020)
$A_y,S_y,Q,I \rightarrow R$	$\gamma$	1/10 $\hbox {day}^{-1}$	SeyedAlinaghi et al. (2021)
$Q \rightarrow I$	$\epsilon$	0.02	Bialek et al. (2020)
$I \rightarrow F$	$\mu$	0.02	Richardson et al. (2020)

**Fig. 4:**
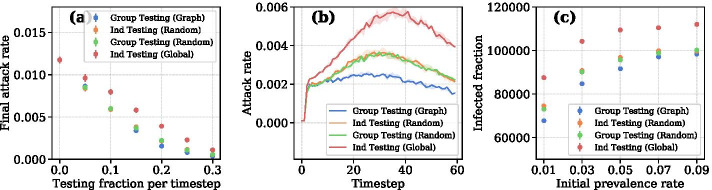
**a** Final attack rate (i.e. attack rate at the last timestep) as a function of testing fraction *q*. $\delta$ is fixed at 0.3. **b** Attack rate over time when $q=0.2$ and $\delta =0.3$. **c** Total case counts at varying levels of initial prevalence

Table 1 shows our model’s epidemiological parameters. Shah et al. (2020) surveys studies of secondary attack rate (SAR) and finds numbers ranging from 4.6% to 35%. (Jing et al. 2020), based on data in Guangzhou, China, finds that SAR may be lower among younger people and gives a 95% confidence interval from 2.4-9.8%. Hence, we selected 9% for the simulation. The secondary attack rate differs from the standard attack rate shown in Fig. 4. SAR refers to the fraction of susceptible agents infected after contact with infected agents. The attack rate is simply the percentage of all susceptible nodes that is infected at a given timestep. In the case of our simulation, SAR distinguishes transmission within the system from imported cases. Though 70% may seem like a high proportion for asymptomatic infections, a study of Rutgers students and medical workers, suggests that students may have a far higher asymptomatic rate than the general population (Barrett et al. 2020).

**Fig. 5:**
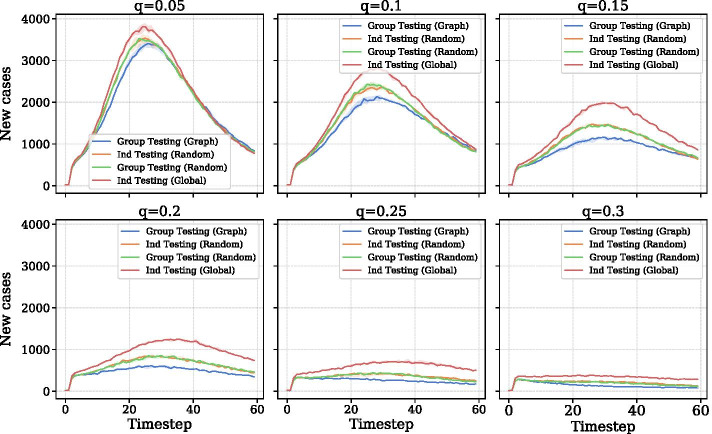
Number of new cases at each timestep for a level of testing *q*. $\delta$ is fixed for all plots at 0.2

### Network model

Based on the SafeGraph data, we construct a network which approximates the movement patterns of a synthetic population. We simulate the movements of around 193,000 agents in 1471 different CBGs across the United States. The total number of links varies between 800,000 to 900,000 depending on the realization due to stochasticity. The network consists of three forms of interaction. First, households within CBGs are modeled by cliques. Household size is assumed to follow a Gaussian distribution. For each CBG, the mean and standard deviation of the distribution is taken from the 2016 ACS. Second, we randomly connect households within a CBG according to a log-normal distribution. Third, agents travel to different CBGs, where the destinations and number of travelers are determined by SafeGraph’s destination_cbgs column (see “Appendix” for definition). If the destination CBG is within our defined set of college towns, these agents will interact with other nodes in the simulation. If the destination is outside the system, nodes are infected with probability $\mu$. We set $\mu =0.0001$ since our focus is on the dynamics within college towns. Further information can be found in the “Appendix”.

Our model roughly follows the hierarchical metapopulation structure detailed by the authors of Watts et al. (2005). Agents are embedded in a hierarchy of successively larger contexts: individuals mix uniformly within a household, form links between households within a CBG according to a log-normal distribution, and travel between CBGs according to destinations derived from the cellphone data.

**Fig. 6:**
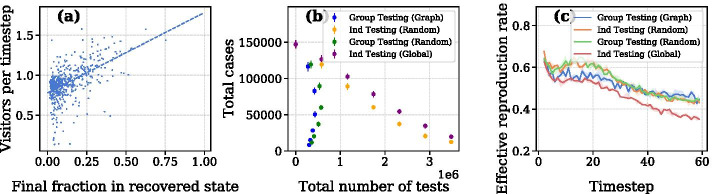
**a** Relationship between a CBG’s mobility and total case count ($q=0.15$ and $\delta =0.2$). Visitors per timestep is normalized by the size of the CBG. **b** Each testing strategy’s total number of tests and resulting case count. **c** Effective reproduction rate *R*(*t*) when $q=0.2$ and $\delta =0.3$

### Simulation

We run the simulation for 60 timesteps, where each timestep corresponds to a day in an epidemic. The initial prevalence rate *p* is 1% for most experiments, though we include a study varying the initial prevalence. At each timestep, we test a fraction *q* of the population. Let *n* represent the total number of agents in the population. We define the four testing strategies below:

- **Individual testing at random**: Ind Testing (Random) chooses a fraction *q* from the population by selecting $q * n$ nodes uniformly at random and test each individually. If a node tests positive, the agent moves into the quarantine state.
- **Individual testing with global rank**: Ind Testing (Global) assigns every node *i* an importance score $s_i$, where $s_i = deg(i) + \lambda * \text {num\_trips}(i)$. $\text {num\_trips(i)}$ refers to the number of trips taken by *i*, updated every timestep, and $\lambda$ is a parameter that controls the relative importance of the two terms. In our experiments, $\lambda$ is set to 0.5. All scores *s* are then normalized so that $\sum _{i=0}^{n} s_i = 1$ and we select a fraction *q* by treating $s_i$ as a probability.
- **Group testing at random**: Group Testing (Random) randomly selects $q * n$ nodes and divides them into pools of 20. We then apply the two-stage group test to each pool.
- **Group testing by network**: Group Testing (Graph) randomly selects $(q * n) / 20$ nodes. For every node *i* in the sample, we construct a pool from neighbors with size $\texttt {max}(deg(i), 20)$ and apply the two-stage group test. The original node *i* is not included in the group test.

Though we focus on the setting where we have limited knowledge of the underlying interaction network, we include the strategy Ind Testing (Global) to study the usefulness of collecting information on the underlying global network.

Though our simulation is not a perfect replica of the real world, there is value in studying testing strategies in a simplified setting. Since we can precisely control epidemiological parameters and track metrics on an individual level, we hope to gain a deeper, mechanistic insights into the dynamics of Covid-19 testing. Previous work has also relied on data-driven simulations to evaluate potential scenarios and vet disease mitigation strategies (Lu et al. 2020; Chang et al. 2021; Deckert et al. 2020).

## Results

We investigate the synthetic model at varying levels of testing fraction *q* and voluntary quarantine parameter $\delta$. For each experiment, we ran ten realizations with different random seeds. We first investigate different levels of testing when voluntary quarantining is fixed ($\delta =0.2$). Figure 5 illustrates the effectiveness of the four testing strategies at varying levels of *q*. When testing is low, all four strategies show the familiar spike in cases around timestep 20. Group testing slightly delays the curve’s peak. As *q* increases, the curve flattens. Group Testing (Graph) demonstrates a marked improvement over the other three methods particularly when $q=0.15$ and $q=0.2$. The marginal benefit of increased testing diminishes sharply after $q=0.2$. At $q=0.3$, daily cases never pass 500 for any method.

We investigate attack rate in Fig. 4a, b. Though there is little variation in the final attack rate, with the exception of Ind Testing (Global), attack rate differs significantly during the course of simulation. Ind Testing (Random) and Group Testing (Random) show nearly identical attack rates, while our method shows a mostly flat attack rate over 60 timesteps.

**Fig. 7:**
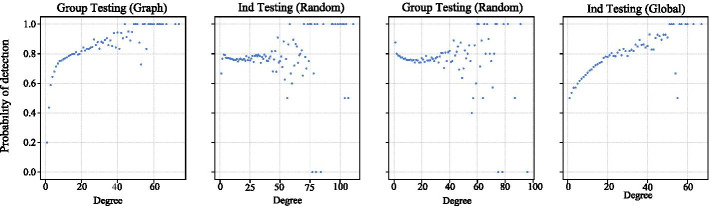
The probability that an infected node is detected as a function of its degree

Surprisingly, Ind Testing (Global) results in the highest number of cases of all four strategies. Though the global strategy has a lower *R*(*t*) as seen in Fig. 6c, we hypothesize this is due to the large number of infections in the first 10 timesteps. After local communities are saturated, infections relative to total infected decline. Nevertheless, the global testing strategy results in higher case counts and attack rates across all levels of *q*. This is likely due to Ind Testing (Global) over-testing the high-degree nodes. Figure 7 shows that global testing has the lowest probability of detecting the nodes with lower degree. Calibrating the strategy to add more randomness to the selection process would likely improve results.

**Fig. 8:**
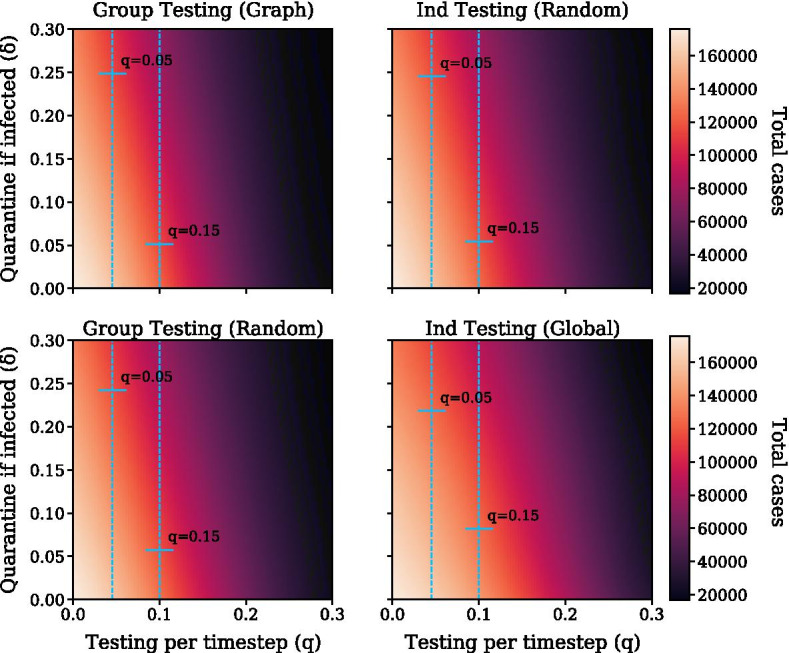
Each figure illustrates the effect of voluntary quarantining ($\delta$) and the degree of testing (*q*) on total COVID cases, averaged across ten trials. For both axes, the experiments are conducted at intervals of 0.05 from 0 to 0.3. Gaussian process regression is used for interpolation. The vertical lines show testing at two different levels, and the horizontal lines show where the two levels of testing result in similar case counts

To evaluate the sensitivity of the testing fraction *q* $\delta$, we ran experiments varying both *q* and $\delta$ at {0, 0.05, 0.1, 0.15, 0.2, 0.25, 0.3}. Figure 8 shows the results of Gaussian process regression for interpolating the number of total cases. For all strategies, increasing *q* has a larger mitigatory effect on disease spread than increasing $\delta$.

For a concrete look at the colleges simulated in our model, Fig. 2 shows case counts over time at the nine colleges with the highest device counts in our sample. These plots are drawn from the scenario where $\delta =0.2$ and $q=0.15$, i.e. approximately 15% of agents are tested at each timestep. In sum, these colleges have 22,162 agents and comprise 11.6% of the total population. Among the nine colleges, Texas A&M University and the University of Alabama show a mostly linear growth in cases. The other universities see a rapid growth in the initial timesteps, with a leveling off after $t=40$. The number of disease cases in a college town is strongly associated with the number of visitors that it receives. We can compute the number of visitors a CBG receives by summing all devices which have that CBG as a destination. Figure 6a illustrates the relationship between the number of visitors, divided by the number of devices within that college town, and the total disease cases. The Pearson correlation between the two metrics is 0.54.

To analyze sensitivity to initial prevalence, we vary initial prevalence from 0.01 to 0.09 at intervals of 0.02. Figure 4c shows that Group Testing (Graph) is robust to variation in the initial prevalence.

We briefly summarize the experimental results with a few observations:

*Group testing requires fewer tests than individual testing to control infection.* From Fig. 6b we see that group testing, in general, requires fewer tests to reduce infection. At a comparable level of infection, group testing (both at random and by network) requires up to 5 times fewer tests than individual testing.

**Fig. 9:**
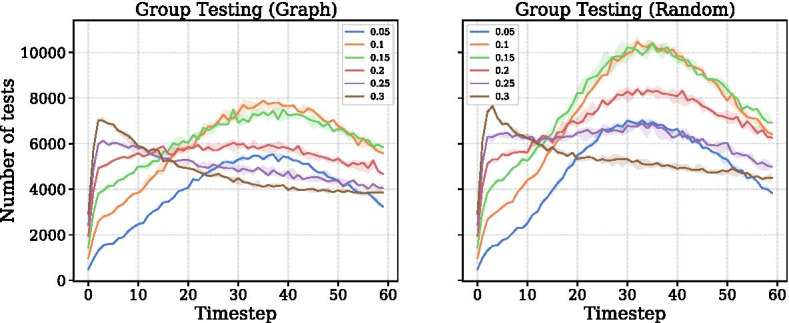
Number of tests over time for group testing strategies at varying levels of testing, where $\delta =0.3$

*Frequent testing is more effective in early stages of an outbreak.* Figure 6b shows that the level of tests needed for group testing is roughly constant at different levels; interestingly, fewer tests can result in fewer cases—a counter-intuitive phenomenon resulting from the *timing* of the tests. The number of group tests varies depending on the prevalence rate at a given time. Figure 9 shows the number of tests at each timestep. When *q* is high, group testing suppresses the outbreak early on and lowers the number of tests necessary at latter timesteps since entire pools will test negative. If *q* is low, the case count rises rapidly at first and leads to additional group tests as pools start to test positive. This holds for both Group Testing (Graph) and Group Testing (Random).

*Testing is most effective when paired with voluntary quarantining.* Figure 8 shows the effect of voluntary quarantine and testing on the total number of cases. As $\delta$ increases to 0.3, cases remain high if $q=0$. When the two are paired together, cases decrease rapidly.

*Group testing by network shows a slight improvement over random group testing in certain settings.* From Fig. 6b, we see that our network-based approach outperforms random group testing by a small amount. Although Group Testing (Graph) generally has smaller pools than the random approach because most randomly selected nodes have less than 20 contacts, it requires fewer tests to mitigate the outbreak. We hypothesize that it has two major advantages over Group Testing (Random). First, the agents within the pool are more likely to be correlated. If they are all connected to a common node, then they are likely mostly uninfected or infected. Second, testing by network allows us to identify the nodes with higher degree early on. Figure 7 shows the probability of detection as a function of an agent’s degree.

*Individual testing by global rank requires careful tuning, unlike group testing.* In our simulation, Ind Testing (Global) underperformed all other testing methods. This is not to say that globally ranking and testing significant nodes is ineffective. Recall how we assign scores to nodes: $s_i = deg(i) + \lambda * \text {num\_trips}(i)$. $\lambda$ must be tuned to properly balance between the two terms, which may be on dramatically different scales. Moreover, $num\_trips$ may not be indicative of a node’s importance, since the agent could be traveling to CBGs with less infection than their home CBG.

## Conclusion

Vaccinations have provided governments and physicians with another tool to combat Covid-19. Yet a limited supply of vaccines, uncertainty about the length of immunity, and the ever-present danger of virus variants mean that testing must remain a core part of our approach to curbing disease spread. Group testing poses significant improvements over individual testing, especially in densely populated environments like college towns where pooling samples is relatively cheap.

In this paper, we proposed a novel method for group testing by network in college towns. Our method suppresses disease outbreaks while requiring fewer tests. Colleges and universities have the unique ability to target testing toward individuals with the highest risk for contracting and spreading Covid-19. Though testing 30% of students every day may seem too high, universities have already begun implementing similar policies. At Harvard University and the University of Illinois Urbana-Champaign during the 2020-2021 term, undergraduates living on campus must take a self-administered Covid-19 test twice a week ($q=0.29$) (Services HUH 2020; U of Illinois Urbana-Champaign 2020). Nevertheless, any targeted testing method must avoid bias and ensure that all students are treated fairly by the administration. Any student or staff member who wants a test should be given reasonable avenues, outside of targeted testing.

Future work in this direction can take advantage of further simulation. As more data becomes available on the effectiveness of vaccines, it would be useful to model vaccination and testing as simultaneous processes. Vaccinations can be incorporated into our model by adding an Immunized ($I_m$) state. Moreover, a greater knowledge of Covid-19’s disease dynamics will improve the predictive power of data-driven simulations (Table 2).

**Table 2:** Abbreviations

Abbreviation	Definition
*N*	Pool size for group test
*x*	Number of groups per pool
*k*	Number of stages per group test
SEIR model	Susceptible-exposed-infected-recovered model
SLIR model	Susceptible-latent-infected-recovered model
i.i.d	independent and identically-distributed
*R*0	Base reproduction number
CBG	Census block group
*q*	Fraction of population tested at each timestep
*n*	Total population
*R*(*t*)	Effective reproduction rate at time *t*
All simulation parameters	See Table 1

## Figures and Tables

**Fig. 10:**
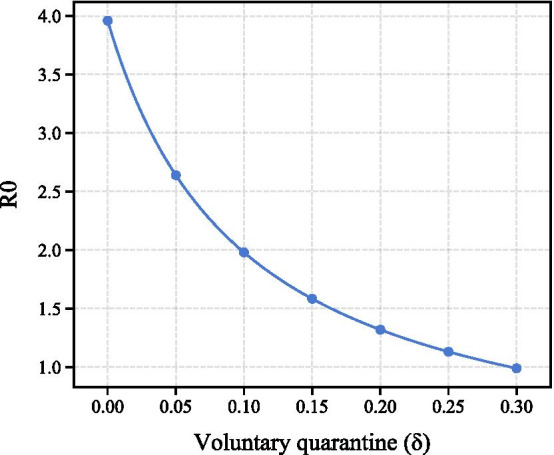
Relationship between $\delta$ and $R_0$

## Data Availability

Code for constructing the mobility network and running simulations is available upon request.

## Appendix

### SafeGraph data

SafeGraph social distancing metrics are based on data going back to January 1, 2019. In an effort to protect the privacy of individuals, differential privacy is applied to all metrics except for device_count.

device_count represents the number of devices whose home is in the census block group (CBG) recorded during the date range. Home, for the purposes of this metric, is “the common nighttime location for the device over a 6 week period where nighttime is 6 pm–7 am.” census_block_groups with count of less than 5 were excluded. Other important schemas for the purposes of this research include median_non_home_dwell_time and destination_cbgs. Median_non_home_dwell_time is an integer value that represents the median dwell time for all devices in device_count for locations outside of the geohash-7 home. Dwell time for each device is calculated by summing the number of minutes spent outside the house throughout the day. destination_cbgs is a JSON with each key being a destination census block group and each value being the number of devices with a home in census_block_group that stopped in the destination census block group for longer than 1 min during the time period. The destination census block group includes the origin_census_block_group.

### Basic reproductive number

In this section we compute the basic reproductive number, $R_0$, based on our parameters. Let x(t) be the fraction of infected individuals, q(t) be the fraction in quarantine, s(t) be the susceptible fraction, and r(t) the recovered fraction.

$$\begin{aligned} \frac{dx}{dt}= & {} \beta s(t) x(t) - \gamma x(t) - \alpha x(t) \\ \frac{dq}{dt}= & {} \alpha x(t) - \gamma q(t) \\ \frac{ds}{dt}= & {} -\beta s(t) x(t) \\ \frac{dr}{dt}= & {} \gamma x(t) + \gamma q(t) \end{aligned}$$

Let $\beta$ be the probability of infection multiplied by the number of average contacts per agent, $\delta$ be the probability of voluntary quarantine after infection, and $\gamma$ be the probability of recovery. In our network, the number of edges ranges between 800,000 and 900,000 due to randomness. Hence, the average degree is around 4.4.

$$\begin{aligned} R_0= & {} \frac{\beta }{\gamma + \delta }\\ R_0= & {} \frac{0.396}{0.1 + \delta } \end{aligned}$$

Figure 10 assumes an entirely susceptible population at $t=0$ and no testing. If $\gamma$ and $\beta$ are fixed, testing is necessary to supplement quarantine measures.

## References

[CR2] Aleta A, Martin-Corral D, y Piontti AP, Ajelli M, Litvinova M, Chinazzi M, Dean NE, Halloran ME, Longini IM, Merler S (2020). Modelling the impact of testing, contact tracing and household quarantine on second waves of Covid-19. Nat Hum Behav.

[CR21] Backer JA, Klinkenberg D, Wallinga J (2020). Incubation period of 2019 novel coronavirus (2019-ncov) infections among travellers from Wuhan, China, 20–28 January 2020. Eurosurveillance.

[CR22] Barrett ES, Horton DB, Roy J, Gennaro ML, Brooks A, Tischfield J, Greenberg P, Andrews T, Jagpal S, Reilly N (2020). Prevalence of sars-cov-2 infection in previously undiagnosed health care workers at the onset of the us Covid-19 epidemic. MedRxiv.

[CR24] Bialek S, Boundy E, Bowen V, Chow N, Cohn A, Dowling N, Ellington S, Covid CD, Team R, COVID C, Team R, COVID C, Team R, et al. (2020) Severe outcomes among patients with coronavirus disease 2019 (Covid-19)—United States, February 12–march 16, 2020. Morbid Mortal Wkly Rep 69(12):343–346 10.15585/mmwr.mm6912e2 PMC7725513 32214079

[CR19] Bureau U.C. (2017) American Community Survey Information Guide. https://www.census.gov/programs-surveys/acs/about/information-guide.html Accessed 5 June 2021

[CR9] Chang S, Pierson E, Koh PW, Gerardin J, Redbird B, Grusky D, Leskovec J (2021). Mobility network models of Covid-19 explain inequities and inform reopening. Nature.

[CR18] Chen MK, Haggag K, Pope DG, Rohla R (2019). Racial disparities in voting wait times: evidence from smartphone data. Rev Econ Stat.

[CR12] Christakis NA, Fowler JH (2010). Social network sensors for early detection of contagious outbreaks. PLoS ONE.

[CR11] Cohen R, Havlin S, ben-Avraham D (2003). Efficient immunization strategies for computer networks and populations. Phys Rev Lett.

[CR4] Crozier A, Rajan S, Buchan I, McKee M (2021). Put to the test: use of rapid testing technologies for Covid-19. BMJ.

[CR10] Deckert A, Bärnighausen T, Kyei NN (2020). Simulation of pooled-sample analysis strategies for Covid-19 mass testing. Bull World Health Organ.

[CR6] Eberhardt JN, Breuckmann NP, Eberhardt CS (2020). Multi-stage group testing improves efficiency of large-scale Covid-19 screening. J Clin Virol.

[CR5] Eis-Hübinger A, Hönemann M, Wenzel J, Berger A, Widera M, Schmidt B, Aldabbagh S, Marx B, Streeck H, Ciesek S, Liebert U, Huzly D, Hengel H, Panninge M (2020). Ad hoc laboratory-based surveillance of sars-cov-2 by real-time rt-pcr using minipools of rna prepared from routine respiratory samples. J Clin Virol.

[CR3] Gollier C, Gossner O (2020). Group testing against Covid-19. Econ Pol Policy Brief.

[CR20] Jing Q, Li Y, Ma M, Gu Y, Li K, Ma Y, Wu D, Wu Y, Luo L, Zhang Z (2020). Contagiousness and secondary attack rate of 2019 novel coronavirus based on cluster epidemics of Covid-19 in Guangzhou. Zhonghua liu xing bing xue za zhi Zhonghua liuxingbingxue zazhi.

[CR17] Klise K, Beyeler W, Finley P, Makvandi M (2021). Analysis of mobility data to build contact networks for Covid-19. PLoS ONE.

[CR13] Levin R, Chao DL, Wenger EA, Proctor JL (2020). Cell phone mobility data reveals heterogeneity in stay-at-home behavior during the sars-cov-2 pandemic. medRxiv.

[CR16] Lu H, Weintz C, Pace J, Indana D, Linka K, Kuhl E (2020). Are college campuses superspreaders? A data-driven modeling study. Comput Methods Biomech Biomed Eng.

[CR15] Paltiel AD, Zheng A, Walensky RP (2020). Assessment of sars-cov-2 screening strategies to permit the safe reopening of college campuses in the United States. JAMA Netw Open.

[CR1] Reyna-Lara A, Soriano-Paños D, Gómez S, Granell C, Matamalas JT, Steinegger B, Arenas A, Gómez-Gardeñes J (2021). Virus spread versus contact tracing: two competing contagion processes. Phys Rev Res.

[CR25] Richardson S, Hirsch JS, Narasimhan M, Crawford JM, McGinn T, Davidson KW, Barnaby DP, Becker LB, Chelico JD, Cohen SL (2020). Presenting characteristics, comorbidities, and outcomes among 5700 patients hospitalized with Covid-19 in the New York City area. JAMA.

[CR8] Salathé M, Kazandjieva M, Lee JW, Levis P, Feldman MW, Jones JH (2010). A high-resolution human contact network for infectious disease transmission. Proc Natl Acad Sci.

[CR28] Services HUH (2020) Viral testing as part of campus reopening. https://huhs.harvard.edu/viral-testing Accessed 5 June 2021

[CR23] SeyedAlinaghi S, Abbasian L, Solduzian M, Yazdi NA, Jafari F, Adibimehr A, Farahani A, Khaneshan AS, Alavijeh PE, Jahani Z (2021). Predictors of the prolonged recovery period in Covid-19 patients: a cross-sectional study. Eur J Med Res.

[CR26] Shah K, Saxena D, Mavalankar D (2020). Secondary attack rate of Covid-19 in household contacts: a systematic review. QJM Int J Med.

[CR29] U of Illinois Urbana-Champaign (2020) On-campus COVID-19 testing. https://covid19.illinois.edu/health-and-support/on-campus-covid-19-testin g/#whoshouldtest Accessed 5 June 2021

[CR14] Walke H, Honein M, Redfield R (2020). Preventing and responding to Covid-19 on college campuses. JAMA.

[CR27] Watts DJ, Muhamad R, Medina DC, Dodds PS (2005). Multiscale, resurgent epidemics in a hierarchical metapopulation model. Proc Natl Acad Sci.

[CR7] Wee S-L, Wang V (2020) Here’s how wuhan tested 6.5 million for coronavirus in days. https://www.nytimes.com/2020/05/26/world/asia/coronavirus-wuhan-tests.htm l. Accessed 5 June 2021

